# Retinopathy of prematurity: Clinical profiles and risk factors

**DOI:** 10.6026/973206300221779

**Published:** 2026-03-31

**Authors:** Sanskriti Ukey, Ankita Baghel, Aditi Mishra, Anamika Dwivedi, Sachin Parmar

**Affiliations:** 1 Department of Ophthalmology, Nand Kumar Singh Chauhan Government Medical College, Khandwa, Madhya Pradesh, India; 2 Department of Ophthalmology, Shyam Shah Medical College, Rewa, Madhya Pradesh, India; 3 Department of Community Medicine, V.K.S. Government Medical College, Neemuch, India

**Keywords:** Retinopathy of Prematurity (ROP), preterm infants, low birth weight, gestational age, Anti-VEGF therapy, screening

## Abstract

Retinopathy of Prematurity (ROP) is a vasoproliferative retinal disease affecting preterm infants due to abnormal oxygen and growth factor regulation, strongly associated with low birth weight and prematurity. This prospective cohort study conducted at Shyam Shah Medical College, Rewa (September 2022 to February 2024) screened 1188 infants with birth weight <2000g and gestational age <37 weeks for ROP assessment. Of the screened infants, 120 (10.1%) were diagnosed with ROP, with Stage 1 and Zone 1 being most prevalent and multiple pregnancies, low birth weight, small for gestational age and oxygen supplementation identified as significant risk factors. Treatment with anti-VEGF injections and laser therapy achieved favorable outcomes in 96.82% of cases. The study emphasizes that early detection through systematic screening and prompt intervention are critical for preventing severe visual impairment in preterm infants.

## Background:

Retinopathy of prematurity (ROP) is a vasoproliferative retinal illness caused by early hyperoxia and late hypoxia interrupting vascular growth and developmental vascularization signaling [1]. Prevention and early detection are necessary. An estimated 32,300 infants become blind or visually handicapped annually due to ROP, with over 65% of cases happening in low- and middle-income countries where limited resources prevent timely screening and intervention [2]. The Early Treatment for Retinopathy of Prematurity (ETROP) trial found that peripheral retinal ablation for high-risk ROP (zone I any stage with plus disease; zone I stage 3 without plus disease; zone II stage 2/3 with plus disease) improved structural and visual outcomes compared to conventional timing, with treatment recommended within 72 hours of Type 1 ROP diagnosis [3]. Meta-analyses show that lower oxygen saturation targets (85-89%) reduce severe ROP but increase mortality compared to higher targets (91-95%), supporting graded saturation strategies. The European Consensus Guidelines recommend saturation targets of 90-94% with alarm limits of 89-95% [4]. Current screening guidelines recommend screening all infants <31 weeks gestational age or <1501g birthweight, using risk-stratified protocols and specialized imaging modalities [5]. Ultra-widefield digital retinal imaging and artificial intelligence-enabled screening are emerging as viable alternatives to traditional binocular indirect ophthalmoscopy, especially in resource-limited settings where telemedicine-based real-time screening is Bevacizumab is superior for zone I stage 3+ ROP, while aflibercept received FDA approval in 2023 and showed efficacy in the FIREFLEYE trial. However, recurrence rates vary (4-23.8% for bevacizumab; up to 83% for other agents), requiring extended surveillance beyond 80 weeks postmenstrual age and careful patient selection [6].

Advanced neonatal ventilation methods such noninvasive high-frequency oscillatory breathing and less invasive surfactant delivery reduce mechanical ventilation duration while preserving ROP safety [7, 8]. Antenatal corticosteroids improve fetal lung maturity and minimize ROP severity [9]. Optimizing oxygen saturation targeting with lower targets in phase I and higher targets from 33 weeks postmenstrual age onward, providing maternal milk and supplemented with docosahexaenoic acid and arachidonic acid reduce severe ROP incidence by 50% [10, 11]. This study increases understanding by pinpointing particular local risk factors. It confirms screening criteria in settings with limited resources. The findings help make protocols well so that fewer people go blind from ROP. The clinical characteristics and risk factors of retinopathy in prematurity.

## Materials and Methods:

The study titled "Assessment of Clinical Profile and Risk Factors of Retinopathy of Prematurity: A Cohort Study in a Tertiary Care Center" was conducted in the Department of Ophthalmology, Shyam Shah Medical College, Rewa (M.P.) from September 2022 to February 2024. This was a prospective cohort study that included both inborn and out born infants requiring universal eye screening for Retinopathy of Prematurity (ROP).

[1] Inclusion criteria: Infants with a birth weight less than 2000g, Infants with a gestational age (GA) of less than 37 weeks, all babies requiring ROP screening as advised by the treating neonatologist.

[2] Exclusion criteria: The study excluded newborns with significant ophthalmic abnormalities that could hinder a clear view of the fundus or make it difficult to perform ROP screening. These included: Microcornea, sclerocornea, corneal opacity, congenital cataract, etc.

## Time of screening:

The initial screening was performed based on the following guidelines:

[1] Between 4 to 6 weeks of postnatal age.

[2] Alternatively, between 31 and 33 weeks' gestational or postmenstrual age, whichever was later?

## Clinical examination:

A thorough clinical examination was conducted by a pediatrician and relevant findings were documented in a standardized proforma.

## The details recorded included:

[1] Baby's name, age, sex, date of birth, gestational age and birth weight.

[2] Information on multiple gestations (e.g., twins, triplets), inborn or out born status.

[3] Size for gestational age (SGA, LGA, or AGA).

[4] Mode of delivery (normal vaginal delivery (NVD) or cesarean section (LSCS)).

[5] Neonatal risk factors such as respiratory distress syndrome (RDS), anaemia, hyperbilirubinemia, shock, intrauterine growth restriction (IUGR), neonatal encephalopathyand low birth weight (LBW), polycythemia, hypoxic-ischemic encephalopathy (HIE), sepsis, oxygen supplementation, phototherapy and any other related diseases.

[6] Presence of any congenital anomalies was also noted.

## Ophthalmic examination:

A comprehensive ophthalmic examination was performed, which included:

[1] Assessment of crossed eyes.

[2] Examination of corneal media clarity.

[3] Pupillary assessment (size and reaction).

[4] Lens evaluation (tunica vasculosa lentis).

[5] Fundus examination after pupil dilation.

## Pupil dilation:

To facilitate the fundus examination, pupils were dilated using Tropicamide 0.5% and Phenylephrine hydrochloride 2.5% eye drops, instilled 30 minutes before the examination. The drops were administered 2-3 times at intervals of 5-10 minutes.

## Instrumentation:

ROP screening was performed using the following instruments:

[1] Binocular indirect ophthalmoscope.

[2] 20D lens.

[3] Pediatric eye speculum.

[4] Pediatric scleral depressor/wire rectus.

[5] Dilating drops (Tropicamide 0.5% and Phenylephrine 2.5%).

[6] Antibiotic drops and Proparacaine eye drops for surface anesthesia.

[7] ROP documentation forms.

[8] Sterile cotton for eye cleaning.

## Fundus examination:

[1] An eye speculum was used to keep the eyes open, followed by surface anesthesia with Proparacaine eye drops.

[2] Fundus examination was conducted using an indirect ophthalmoscope with a +20.00 aspheric lens.

[3] Both eyes were examined, with special attention paid to the temporal retina.

## ROP classification:

The findings of the fundus examination were documented according to the International Classification of Retinopathy of Prematurity (ICROP) Stage and Zone classification system:

## Zones of ROP:

[1] Zone 1: A circle with a radius twice the estimated distance is connecting the optic disc to the foveal center.

[2] Zone 2: A circular region that spans nasally from the outer limit of Zone 1 to the nasal oraserrata.

[3] Zone 3: The crescent of peripheral retina that extends beyond Zone 2.

## Stages of ROP:

[1] Stage 1: A flat, thin, white line dividing vascularized and avascular areas.

[2] Stage 2: A ridge with height, width and volume.

[3] Stage 3: A ridge with extraretinal fibrovascular growth infiltrating the vitreous.

[4] Stage 4: Partial retinal detachment (A: extrafoveal, B: retinal detachment including the fovea).

[5] Stage 5: Total retinal detachment (A: optic nerve head visible, B: optic nerve head not visible).

## Plus disease:

Defined by greater venous dilatation or arterial tortuosity than usual but insufficient for plus disease diagnosis

## Aggressive ROP (AROP):

A severe form with posterior location and significant plus disease

## Treatment:

The treatment decisions were based on the ETROP (Early Treatment for Retinopathy of Prematurity) guidelines, addressing severe ROP, including:

[1] Type 1 ROP: Any stage in Zone I with plus disease, or Stage 3 in Zone I without plus disease.

[2] Type 2 ROP: Zone I, Stage 1 or 2 ROP without plus disease, or Zone II, Stage 3 ROP without plus disease.

## Follow-up:

Follow-up screenings were conducted at the following intervals:

[1] 1 week or less: Zone I ROP Stage 1 or 2 suspected AROP or Stage 3 ROP in Zone I.

[2] 1-2 weeks: Zone II, Stage 2 ROP or unequivocally regressing ROP.

[3] 2 weeks: Zone II, Stage 1 ROP or unequivocally regressing ROP.

[4] 2-3 weeks: Zone III, Stage 1 or 2 ROP or regressing ROP.

## Retinal screenings were stopped when:

[1] Full retinal vascularization was achieved.

[2] Postmenstrual age of 45 weeks with no Type 1 ROP or more severe ROP.

[3] Complete regression of ROP.

## Outcome assessment:

The outcome was classified at 3 months as:

[1] Favorable outcome: Full regression of ROP with a flat retina.

[2] Unfavorable outcome: Advancement to Stage 4A, 4B, Stage 5, falciform fold, or central media opacity that prevents retinal inspection.

## Statistical analysis:

Descriptive statistics were used to summarize the data, with the incidence rate of ROP calculated as a proportion. The association of various risk factors with ROP was studied by comparing their presence in infants with and without ROP, using the chi-square test. Statistical significance was defined as a p-value less than 0.05.Data was compared with 2015 data regarding incidence rate, risk factors, clinical trends and outcomes.

## Results and Discussion:

The cohort of the study included 1,188 babies who were tested on retinopathy of prematurity, among which 719 males (61) and 469 females (39) were born (Table 1 - see PDF). The general prevalence rate of ROP was 10.1% (120/1,188) with a significant negative correlation between gestational age and ROP prevention; babies born at ≤28 weeks experienced the highest prevalence rate of 46.15% whereas those born above 36 weeks had the lowest prevalence rate of 0.44% (Table 5 - see PDF). The mean birth weight (1.4 kg vs. 2.4 kg in the ROP and no ROP respectively) and the mean gestational age (32 weeks vs. 36.7 weeks in the ROP and no ROP respectively) were significantly different between the two groups (Tables 2 and 4 - see PDF). The number of babies was distributed as the majority of them (684, 57.6) were born during the period of >36 gestational weeks, with the number of babies decreasing with the lower gestational age bracket (Table 3 - see PDF). The risk factors analysis showed that multiple pregnancy (20.83% vs. 2.99, p<0.001), small-for-gestational-age (47.5% vs. 12.64, p<0.001), respiratory distress syndrome (46.6% vs. 19.56, p<0.001), oxygen supplementation (90% vs. 43.91, p<0.001) and low birth weight (97.5% vs. 4 Female gender was marginally yet meaningfully related to ROP (48.33% vs. 38.38, p=0.042) (Table 6 - see PDF).

The current ROP incidence of 22% in preterm infants corresponds well with the results of Vinekar *et al.* [12] and Blazon *et al.* [13] who found ROP rates of 20-30% in Indian neonatal intensive care units, especially highlighting the susceptibility of extremely preterm groups. This cohort of predominantly male babies (61%), conforms to epidemiological evidence by Dhaliwal *et al.* [14] Suchandra *et al.* [15] Akkawi *et al.* [16] and Nayyar *et al.* [17] are showed that a male preponderance existed in Indian ROP populations; Mukherjee, 374 (54.1%) babies were male similarly Salma *et al.* [18] and Sheth *et al.* [19] showed that a male preponderance existed in non-Indian 52% this indicates possible sex-linked predisposing factors that should be researched. The birth weight versus ROP risk ratio, whereby, the affected infants had a median birth weight of 1.4 kg compared to the unaffected babies of 2.4 kg, is also similar to the results of Fortes Filho *et al.* [20] who found low birth weight to be the strongest predictor of severe ROP across various populations. Similarly, Shah *et al.* [21] strongest predictor of severe ROP among Indian population. Equally, the gestational age distribution with the greatest ROP rates 28 weeks (46.15) 28-30 weeks (43.90) also confirms findings of Blencowe *et al.* [22] who reported exponentially rising risks of ROP with decreasing gestational age in their worldwide systematic review. Identifying the factors of multiple pregnancies, small for gestational age, oxygen supplement administration and respiratory distress syndrome as the main risk factors are consistent with the complete meta-analysis by Good *et al.* [23] that defined these variables as stable ROP predictors in a variety of healthcare contexts. The shock protective effect observed in this study is an interesting observation that appears contrary to common patterns of hemodynamic risk reported by Shah *et al.* [24] hence warranting further mechanistic research. Therapeutic outcomes of 96.82% positive response to anti-VEGF therapy are comparable to the outcomes of Mintz-Hittner *et al.* [25] whose BEAT-ROP was able to set the bevacizumab efficacy as a zone I and posterior zone II ROP. This 100 percent success rate using laser therapy in this cohort is higher than that achieved by Good *et al.* [23] in the ETROP study and may be due to better selection of cases and technical skills of modern practice. The fact that, the incidence of ROP has increased by 33.33% in 2015 but now by 22.9% in the current study indicates that neonatal care practices are now safer, which is in line with the trends reported by Bas *et al.* [26] who reported similar decrease in the severity of ROP after the introduction of standardized oxygen monitoring protocols. The higher frequency of aggressive posterior ROP compared with staged disease in this cohort is indicative of diagnostic refinements as outlined by International Classification of ROP Revisited Group and the importance of identifying this rapidly progressive phenotype promptly to intervene [27].

## Conclusion:

The significant role of gestational age, birth weight and other risk factors such as oxygen supplementation and multiple gestations in the development of ROP is shown. The incidence of ROP was higher in preterm babies, particularly those with lower gestational age and birth weight. The use of intravitreal Anti-VEGF and laser therapy showed promising outcomes, with most babies experiencing favorable results. Compared to previous studies, this cohort showed a lower incidence of ROP, which may be attributed to improved neonatal care and screening practices. Thus, we show the importance of early screening, timely intervention and monitoring of at-risk infants to reduce the burden of ROP and prevent vision loss.
